# Supplementary material for: Th17/Regulatory T-Cell Imbalance and Acute Kidney Injury in Patients with Sepsis
Source: J Clin Med. 2022 Jul 12;11(14):4027. doi: 10.3390/jcm11144027 (PMC9320223; doi:10.3390/jcm11144027)
Supplement: Supplementary file 1 [file jcm-11-04027-s001.zip › jcm-1775549-supplementary.pdf]

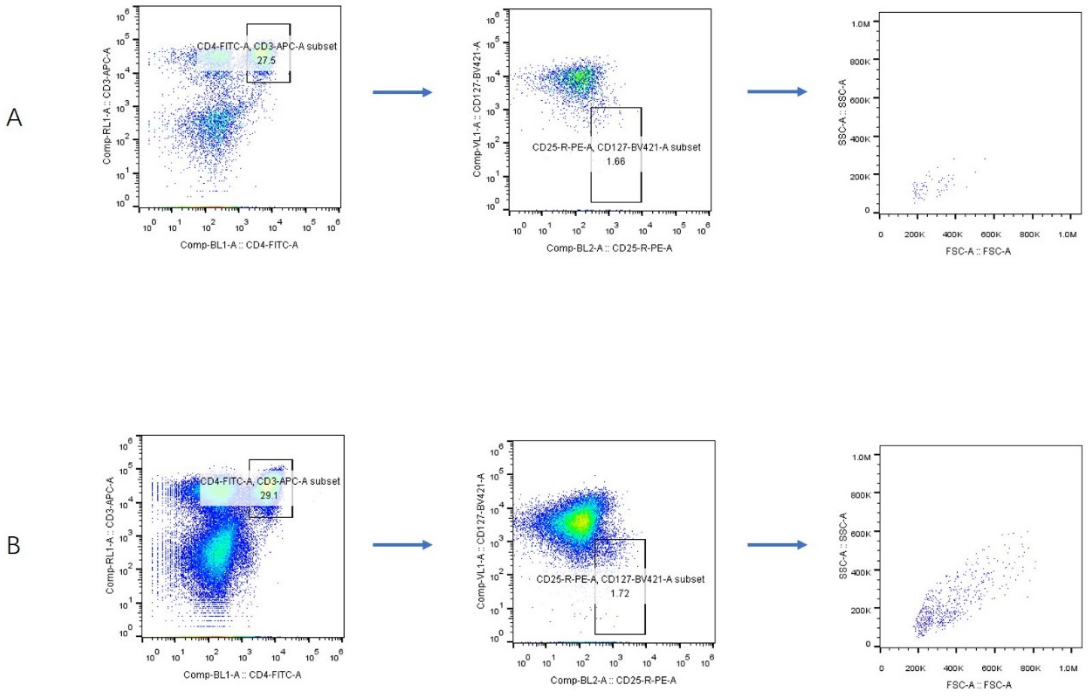

Figure S1. Flow cytometry analysis for Treg population in SAKI group (**A**) and sepsis-without-acute kidney injury group (**B**).

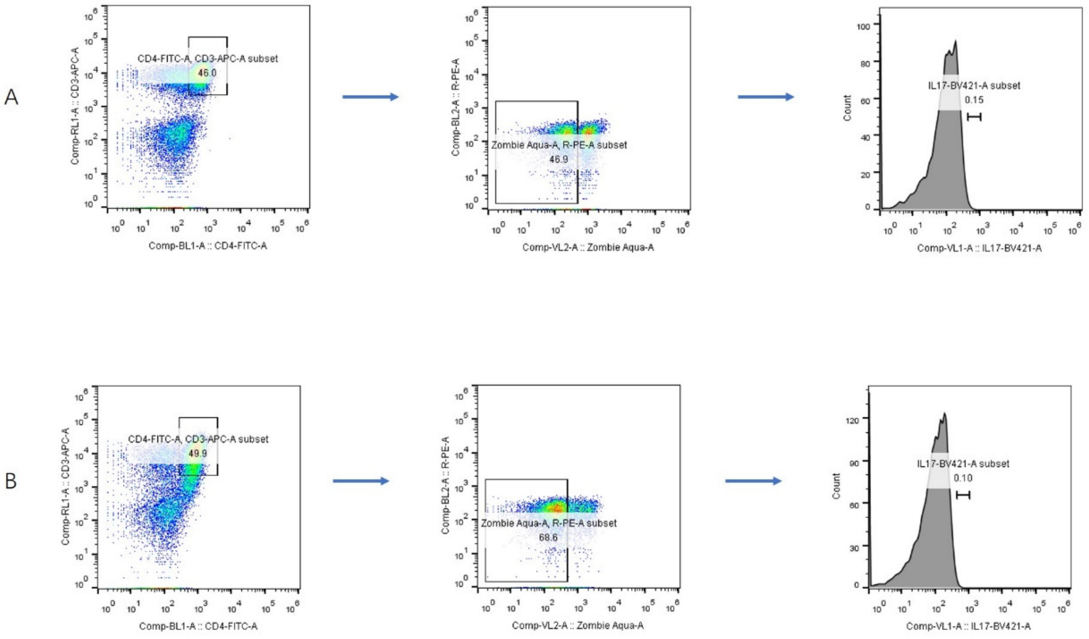

Figure S2. Flow cytometry analysis for Th17 population in SAKI group (**A**) and sepsis-without-acute kidney injury group (**B**).

Table S1. Comparison of clinical characteristics between SAKI and sepsis-without-AKI groups after exclusion of patients with gastrointestinal infection.

	**Total (*n* = 120)**	**SAKI Group (*n* = 56)**	**Sepsis-without-AKI Group (*n* = 64)**	**Test Value**	***P* Value**
Male [*n* (%)]	70 (58.3)	38 (67.9)	32 (50.0)	3.918	0.048
Age—year (median [Q1,Q2])	68.00 (55.00, 75.00)	69.50 (55.00, 79.00)	67.50 (56.25,74.50)	−0.442	0.658
BMI—kg/m^2 (mean ± SD)	23.71 ± 4.81	23.44 ± 4.81	23.95 ± 4.84	−0.610	0.542
Comorbidities					
Hypertension [*n* (%)]	74 (61.7)	40 (71.4)	34 (53.1)	4.233	0.040
Diabetes [*n* (%)]	36 (30.0)	22 (39.3)	14 (21.9)	4.311	0.038
CKD [*n* (%)]	20 (16.7)	10 (17.9)	10 (15.6)	0.107	0.743
Chronic cardiovascular disease [*n* (%)]	50 (41.7)	26 (46.4)	24 (37.5)	0.980	0.322
Chronic lung disease [*n* (%)]	16 (13.3)	10 (17.9)	6 (9.4)	1.860	0.173
Chronic liver disease [*n* (%)]	8 (6.7)	2 (3.6)	6 (9.4)	1.617	0.204
Nervous system disease [*n* (%)]	36 (30.0)	14 (25.0)	22 (34.4)	1.250	0.264
Rheumatic system disease [*n* (%)]	4 (3.3)	2 (3.6)	2 (3.1)	0.018	0.892
Malignant tumor [*n* (%)]	38 (31.7)	16 (28.6)	22 (34.4)	0.465	0.495
Physiological parameters					
SOFA score (median [Q1,Q2])	6.50 (3.25, 8.00)	8.00 (6.00, 11.00)	4.50 (3.00, 7.00)	−5.240	0.000
APACHEII score (median [Q1,Q2])	20.00 (15.00, 25.00)	20.50 (14.25, 25.00)	20.00 (15.00, 25.00)	−0.084	0.933
Septic shock [*n* (%)]	80 (66.7)	42 (75.0)	38 (59.4)	3.281	0.070
Numbers of organ dyfunctions caused by infection (median [Q1,Q2])	3 (2,5)	4 (3,5)	2 (1,3)	−6.091	0.000
Site of infection					
respiratory system [*n* (%)]	58 (48.3)	22 (39.3)	36 (56.3)	3.442	0.064
urinary system [*n* (%)]	20 (16.7)	18 (32.1)	2 (3.1)	18.107	0.000
biliary tract [*n* (%)]	6 (5.0)	4 (7.1)	2 (3.1)	1.015	0.314
Thoracic and abdominal cavity [*n* (%)]	84 (70.0)	32 (57.1)	52 (81.3)	8.265	0.004
bloodstream [*n* (%)]	4 (3.3)	2 (3.6)	2 (3.1)	0.018	0.892
skin and soft tissue [*n* (%)]	2 (1.7)	0 (0)	2 (3.1)	1.780	0.182
central nervous system [*n* (%)]	4 (3.3)	0 (0)	4 (6.3)	3.621	0.057
NAGL—ng/ mL(median [Q1,Q2])	60.02 (53.18, 75.10)	74.27 (60.25, 81.63)	54.68 (51.06, 61.93)	−5.103	0.000
Urine output—L/24 h (median [Q1,Q2])	1.85 (1.19, 2.57)	1.63 (0.40, 2.53)	2.02 (1.37, 2.67)	−2.609	0.009
Cr—umol/L (median [Q1,Q2])	78.65 (59.12, 124.77)	123.65 (92.80, 204.75)	63.70 (50.28, 74.13)	−7.871	0.000
BUN—mmol/L (median [Q1,Q2])	10.02 (5.91, 15.43)	14.38 (10.76, 20.58)	6.34 (4.38, 8.42)	−6.313	0.000
PCT—ng/ mL(median [Q1,Q2])	2.91 (0.69, 19.52)	19.20 (4.85, 34.11)	1.31 (0.55, 4.44)	−5.669	0.000

Lac—mmol/L (median [Q1,Q2])	1.50 (1.02, 1.97)	1.80 (1.12, 3.30)	1.40 (1.00, 1.83)	−2.929	0.003
OI (median [Q1,Q2])	248.00 (180.00, 341.75)	236.00 (183.00, 340.00)	257.00 (178.25, 325.75)	−0.821	0.412
WBC—x10^9/L (median [Q1,Q2])	11.56 (8.17, 16.71)	11.72 (7.99, 22.17)	12.02 (8.28, 14.59)	−1.157	0.247
GR—x10^9/L (median [Q1,Q2])	10.29 (6.86, 15.03)	10.84 (7.07, 20.02)	10.41 (6.78, 12.92)	−1.599	0.110
LY—x10^9/L (median [Q1,Q2])	0.71 (0.47, 1.07)	0.69 (0.45, 1.10)	0.87 (0.54, 1.13)	−0.968	0.333
NLR (median [Q1,Q2])	13.31 (8.71, 22.15)	16.06 (11.07, 32.77)	11.80 (7.71, 18.40)	−2.841	0.004
Immune indexes					
T-lymphocyte ratio—% (median [Q1,Q2])	69.01 (62.90, 80.07)	68.95 (51.38, 76.82)	70.59 (62.91, 83.97)	−1.089	0.276
CD^{4+} T-lymphocyte ratio—% (median [Q1,Q2])	41.63 (27.25, 51.06)	40.22 (28.11, 50.68)	42.79 (26.69, 51.18)	−0.151	0.880
Treg cell ratio—% (median [Q1,Q2])	1.39 (0.69, 2.23)	1.33 (0.56, 2.27)	1.49 (0.86, 2.23)	−0.705	0.481
Th17 cell ratio—% (median [Q1,Q2])	0.13 (0.07, 0.23)	0.15 (0.12, 0.24)	0.09 (0.06, 0.19)	−3.463	0.001
Th17/Treg ratio (median [Q1,Q2])	0.09 (0.05, 0.23)	0.13 (0.07, 0.33)	0.06 (0.05, 0.16)	−3.304	0.001
IL10—pg/ mL(median [Q1,Q2])	25.46 (10.56, 59.50)	32.00 (21.19, 101.47)	14.48 (7.41, 37.94)	−4.230	0.000
IL17—pg/ mL(median [Q1,Q2])	2.62 (0.98, 5.77)	4.29 (1.95, 11.56)	1.71 (0.70, 3.32)	−4.085	0.000
TNF—a—pg/ mL(median [Q1,Q2])	6.33 (3.95, 10.54)	7.74 (2.89, 12.59)	5.59 (3.95, 10.55)	−0.118	0.906
General outcomes					
28—day mortality [*n* (%)]	19 (15.8)	9 (16.1%)	10 (15.6)	0.004	0.947
Hospital length of stay—days (median [Q1,Q2])	15.00 (9.25, 39.50)	15.50 (8.00, 38.75)	14.50 (10.00, 39.50)	−0.717	0.474
In ICU length of stay— days (median [Q1,Q2])	9.50 (6.00, 17.00)	8.50 (7.00, 17.00)	10.00 (5.00, 17.50)	−0.538	0.591
Expenses in ICU—CNY ten thousand (median [Q1,Q2])	6.54 (3.46, 14.06)	7.89 (3.65, 16.42)	5.69 (3.46, 13.64)	−1.189	0.234
Treatments during hospitalization					
ventilator [*n* (%)]	70 (58.3)	34 (60.7)	36 (56.3)	0.245	0.621
vasoactive drugs [*n* (%)]	74 (61.7)	40 (71.4)	34 (53.1)	4.233	0.040
blood transfusion [*n* (%)]	34 (28.3)	24 (42.9)	10 (15.6)	10.908	0.001

BMI, body mass index; CKD, chronic kidney diseases; SOFA, Sequential Organ Failure Assessment; APACHE II, Acute Physiology and Chronic Health Evaluation 2; NAGL, neutrophil gelatinase-associated lipocalin; Cr, serum creatinine; BUN, blood urea nitrogen; PCT, procalcitonin; Lac, lactic acid; OI, oxygenation index; WBC, white blood cell; GR, neutrophil granulocyte; LY, lymphocyte; NLR, neutrophil/lymphocyte ratio; Th17, helper T cell 17; Treg, regulatory T cell; IL-10, Interleukin-10; IL-17, Interleukin-17; TNF-α, Tumor necrosis factor alpha; AKI, acute kidney injury; ICU, intensive care unit.

Table S2. Risk factor analysis for the occurrence of SAKI after exclusion of patients with gastrointestinal infection.

	Univariable Logistic Regression		Multivariable Logistic Regression	
	**Odds Ratio (95% CI)**	***p* Value**	**Odds Ratio (95% CI)**	***p* Value**
Male	0.47 (0.23–0.99)	0.049		
Hypertension	2.21 (1.03–4.71)	0.041		
Diabates	2.31 (1.03–5.13)	0.040		
SOFA	1.48 (1.26–1.73)	0.000	1.35 (1.08–1.69)	0.008
Infection of urinary system	14.68 (3.23–66.85)	0.001		
Infection of thoracic and abdominal cavity	0.31 (0.14–0.69)	0.005		
Numbers of organ dysfunctions caused by infection	2.24 (1.72–3.25)	0.000		
Use of vasoactive drugs	2.21 (1.03–4.72)	0.041		
Use of blood transfusion	4.05 (1.72–9.55)	0.001		
NAGL	1.06 (1.04–1.09)	0.000	1.08 (1.04–1.14)	0.000
Urine output	0.62 (0.43–0.87)	0.006		
Cr	1.04 (1.02–1.05)	0.000		
BUN	1.31 (1.18–1.45)	0.000		
PCT	1.09 (1.05–1.14)	0.000	1.09 (1.04–1.15)	0.001
Lac	1.33 (1.04–1.69)	0.023		
NLR	1.01 (0.99–1.03)	0.208		
Th17	90.36 (3.42–2383.83)	0.007		
IL-10	1.00 (1.00–1.01)	0.027	0.99 (0.98–1.00)	0.235
IL-17	1.13 (1.05–1.23)	0.002	1.09 (0.98–1.22)	0.098
Th17/Treg ratio	59.85 (3.57–1003.73)	0.004	1071.33 (1.89–606,477.72)	0.031

SAKI, sepsis-induced acute kidney injury; CI, confidence interval; SOFA, Sequential Organ Failure Assessment; NAGL, neutrophil gelatinase-associated lipocalin; Cr, serum creatinine; BUN, blood urea nitrogen; PCT, procalcitonin; Lac, lactic acid; NLR, neutrophil/lymphocyte ratio; Th17, helper T cell 17; IL-10, Interleukin-10; IL-17, Interleukin-17.

Table S3. Comparison of clinical characteristics between AKI stage 3 and AKI stage 1 or 2 in the subgroup of SAKI patients.

	**Total (*n* = 60)**	**KDIGO Stage3 Group (*n* = 32)**	**Non-KDIGO Stage3 Group (*n* = 28)**	**Test Value**	***p* Value**
Male [*n* (%)]	38 (63.3)	22 (68.8)	16 (57.1)	0.866	0.352
Age—year (median [Q1, Q2])	67.50 (54.00,76.00)	68.50 (51.75, 71.25)	67.50 (55.00, 83.00)	−0.712	0.476
BMI—kg/m² (mean ± SD)	23.44 ± 4.76	21.94 ± 3.92	25.15 ± 5.12	2.742	0.008
Comorbidities					
Hypertension [*n* (%)]	40 (66.7)	18 (56.3)	22 (78.6)	3.348	0.067
Diabetes [*n* (%)]	22 (36.7)	8 (25.0)	14 (50.0)	4.019	0.045
CKD [*n* (%)]	10 (16.7)	6 (18.8)	4 (14.3)	0.214	0.643
Chronic cardiovascular disease [*n* (%)]	26 (43.3)	14 (43.8)	12 (42.9)	0.005	0.944
Chronic lung disease [*n* (%)]	10 (16.7)	6 (18.8)	4 (14.3)	0.214	0.643
Chronic liver disease [*n* (%)]	2 (3.3)	2 (6.3)	0	1.801	0.178
Nervous system disease [*n* (%)]	14 (23.3)	6 (18.8)	8 (28.6)	0.805	0.370

Rheumatic system disease [*n* (%)]	2 (3.3)	2 (6.3)	0	1.801	0.178
Malignant tumor [*n* (%)]	16 (26.7)	8 (25.0)	8 (28.6)	0.097	0.755
Physiological parameters					
SOFA score (median [Q1, Q2])	8.00 (6.00, 11.00)	9.50 (7.00, 11.75)	7.50 (6.00, 10.00)	−2.057	0.040
APACHEII score (median [Q1, Q2])	20.50 (15.00, 25.00)	21.50 (16.00, 28.75)	18.00 (14.00, 25.00)	−1.396	0.163
Septic shock [*n* (%)]	46 (76.7)	26 (81.3)	20 (71.4)	0.805	0.370
Numbers of organ dysfunctions caused by infection (median [Q1, Q2])	4 (3,5)	5 (4,5)	4 (3,5)	−2.130	0.033
Site of infection					
respiratory system [*n* (%)]	22 (36.7)	12 (37.5)	10 (35.7)	0.021	0.886
urinary system [*n* (%)]	18 (30.0)	8 (25.0)	10 (35.7)	0.816	0.366
gastrointestinal [*n* (%)]	4 (6.7)	4 (12.5)	0	3.750	0.053
biliary tract [*n* (%)]	4 (6.7)	2 (6.3)	2 (7.1)	0.019	0.890
Thoracic and abdominal cavity [*n* (%)]	32 (53.3)	18 (56.3)	14 (50.0)	0.234	0.628
bloodstream [*n* (%)]	2 (3.3)	0	2 (7.1)	2.365	0.124
NAGL—ng/ mL(median [Q1, Q2])	74.10 (60.01, 80.94)	74.10 (56.81, 79.91)	73.81 (60.25, 85.33)	−0.148	0.882
Urine output—L/24 h (median [Q1, Q2])	1.48 (0.39, 2.28)	0.46 (0.11, 1.63)	2.26 (1.35, 2.86)	−4.625	0.000
Cr—umol/L (median [Q1, Q2])	127.00 (98.20, 206.10)	133.20 (100.40, 342.50)	121.35 (86.90, 155.50)	−1.423	0.155
BUN—mmol/L (median [Q1, Q2])	13.38 (10.04, 20.48)	15.43 (11.00, 26.26)	11.56 (7.50, 18.41)	−2.312	0.021
PCT—ng/ mL(median [Q1, Q2])	19.06 (3.30, 33.84)	19.40 (4.06, 27.49)	15.76 (2.79, 34.11)	−0.124	0.901
Lac—mmol/L (median [Q1, Q2])	1.80 (1.20, 3.10)	2.05 (1.33, 4.80)	1.55 (1.10, 2.10)	−2.314	0.021
OI (median [Q1, Q2])	236.00 (172.00, 345.00)	216.00 (149.25, 371.00)	258.00 (180.00, 341.00)	−0.534	0.594
WBC—$\times 10^9$/L (median [Q1, Q2])	11.72 (7.93, 23.34)	13.84 (6.77, 25.13)	11.15 (8.17, 16.23)	−0.889	0.374
GR—$\times 10^9$/L (median [Q1, Q2])	10.84 (6.97, 20.95)	12.54 (5.51, 23.75)	9.92 (7.46, 15.29)	−1.008	0.313
LY—$\times 10^9$/L (median [Q1, Q2])	0.70 (0.46, 1.11)	0.70 (0.50, 1.28)	0.72 (0.36, 1.11)	−1.097	0.273
NLR (median [Q1, Q2])	16.06 (10.82, 31.70)	15.84 (7.61, 33.75)	16.21 (11.82, 30.58)	−0.237	0.813
Immune indexes					
T-lymphocyte ratio—% (median [Q1, Q2])	68.81 (55.49, 75.65)	68.81 (62.22, 77.58)	69.38 (42.49, 75.65)	−0.593	0.553
CD^{4+}-Tlymphocyte ratio—% (median [Q1, Q2])	39.72 (27.88, 49.54)	39.35 (31.61, 53.04)	41.15 (21.37, 49.54)	−1.008	0.313
Treg cell ratio—% (median [Q1, Q2])	1.33 (0.59, 2.32)	1.46 (0.67, 2.56)	1.23 (0.55, 1.51)	−1.156	0.247
Th17 cell ratio—% (median [Q1, Q2])	0.14 (0.11, 0.24)	0.16 (0.11, 0.25)	0.14 (0.11, 0.23)	−0.682	0.495
Th17/Treg ratio (median [Q1, Q2])	0.11 (0.07, 0.28)	0.13 (0.08, 0.25)	0.10 (0.06, 0.36)	−0.267	0.790

IL10—pg/ mL(median [Q1, Q2])	35.55 (25.46, 109.01)	55.47 (27.29, 258.77)	27.31 (14.61, 71.00)	−2.283	0.022
IL17—pg/ mL(median [Q1, Q2])	4.87 (2.03, 12.02)	6.47 (2.55, 12.17)	3.60 (1.28, 7.39)	−1.841	0.066
TNF-α—pg/ mL(median [Q1, Q2])	8.51 (4.05, 12.98)	8.51 (2.51, 10.25)	9.85 (4.62, 19.89)	−1.239	0.215
General outcomes					
28—day mortality [*n* (%)]	10 (16.7)	6 (18.8)	4 (14.3)	0.214	0.643
Hospital length of stay days (median [Q1, Q2])	15.50 (8.00, 32.00)	22.50 (10.25, 41.00)	11.00 (8.00, 27.00)	−1.248	0.212
In ICU length of stay days (median [Q1, Q2])	9.50 (7.00, 17.00)	15.50 (7.25, 30.50)	8.00 (7.00, 10.00)	−2.681	0.007
Expenses in ICU—CNY ten thousand (median [Q1, Q2])	7.89 (4.03, 17.83)	11.52 (5.15, 22.71)	4.95 (3.00, 10.06)	−3.202	0.001
Treatments during hospitalization					
ventilator [*n* (%)]	36 (60.0)	22 (68.8)	14 (50.0)	2.188	0.139
vasoactive drugs [*n* (%)]	44 (73.3)	28 (87.5)	16 (57.1)	7.037	0.008
renal replacement therapy [*n* (%)]	26 (43.3)	24 (75.0)	2 (7.1)	28.003	0.000
Blood transfusion [*n* (%)]	28 (46.7)	22 (68.8)	6 (21.4)	13.436	0.000

BMI, body mass index; CKD, chronic kidney diseases; SOFA, Sequential Organ Failure Assessment; APACHE II, Acute Physiology and Chronic Health Evaluation 2; NAGL, neutrophil gelatinase-associated lipocalin; Cr, serum creatinine; BUN, blood urea nitrogen; PCT, procalcitonin; Lac, lactic acid; OI, oxygenation index; WBC, white blood cell; GR, neutrophil granulocyte; LY, lymphocyte; NLR, neutrophil/lymphocyte ratio; Th17, helper T cell 17; Treg, regulatory T cell; IL-10, Interleukin-10; IL-17, Interleukin-17; TNF-α, Tumor necrosis factor alpha; AKI, acute kidney injury; ICU, intensive care unit.
